# Analysis of Quality Normative Requirements: Contributions to the Alignment and Standardization of Standards Applied in Health Laboratories

**DOI:** 10.3390/biotech15030069

**Published:** 2026-08-19

**Authors:** Saada L. C. Fernandez, Katharine V. S. Hodel, Renata Almeida de Souza, Hayna Malta-Santos, Camila Duarte Ferreira Ribeiro, Bruna A. S. Machado

**Affiliations:** 1 SENAI CIMATEC, Postgraduate Program in Industrial Management and Technology, SENAI CIMATEC University, Salvador 41650-010, BA, Brazil; saada.fernandez@fiocruz.br (S.L.C.F.); hayna.santos@fbter.org.br (H.M.-S.); 2 Oswaldo Cruz Foundation—Fiocruz, Rio de Janeiro 21040-900, RJ, Brazil; renata.almeida@fiocruz.br; 3 SENAI CIMATEC, SENAI Institute of Innovation (ISI) in Health Advanced Systems (CIMATEC ISI SAS), SENAI CIMATEC University, Salvador 41650-010, BA, Brazil; katharine.hodel@fieb.org.br; 4 Nutrition School, Federal University of Bahia, Salvador 40110-907, BA, Brazil; camiladuartef@ufba.br; 5 Graduate Program in Food Science, Faculty of Pharmacy, Federal University of Bahia, Salvador 40170-290, BA, Brazil

**Keywords:** standards, requirements, quality management, health laboratories

## Abstract

The implementation of Quality Management Standards (QMS) in health laboratories is essential to ensuring the traceability, reliability, and reproducibility of results, thus supporting compliance and continuous improvement. This study identifies and compares the requirements adopted in four widely used four standards for health laboratoriesto evaluate their equivalence and contribute to the harmonization of normative terminology. A comparative analysis of the requirements and their applications in health laboratories was conducted. Subsequently, interrelationships and equivalence among the standards were assessed, followed by an evaluation of the terminology used and the grouping of requirements across the four normative documents. The results revealed a substantial overlap among the standards, particularly regarding quality management, risk management, personnel competence, documentation control, and continuous improvement. However, differences in terminology, structure, and scope were identified, which may hinder the uniform interpretation and implementation of quality systems. The harmonization of terms and equivalent requirements could facilitate compliance, integration of management systems, and accreditation processes. The findings demonstrate that standardizing normative terminology contributes to greater alignment among quality standards, improving their understanding, dissemination, and application in health laboratories while supporting more efficient and reliable quality management practices.

## 1. Introduction

Health laboratories play a crucial role in maintaining and promoting public and individual health. The importance of these laboratories can be understood through accurate and rapid diagnosis in disease monitoring, research and development, quality control, prevention, and (among other actions) support for public health programs [1].

Standardizing requirements for implementing Quality Management Systems (QMS) through specific standards for health laboratories ensures the consistency, reliability, and safety of the services offered by these establishments [2,3,4,5]. In this context, four of the most relevant standards are as follows: ABNT NBR ISO 9001:2015, ABNT NBR ISO/IEC 17025:2017, ABNT NBR ISO 15189:2015, and NIT DICLA 035:2019 [6,7,8,9].

Among the standards considered in this study, ISO 9001 provides a generic framework for Quality Management Systems (QMS) applicable to organizations of any size or sector, emphasizing customer focus, process management, risk-based thinking, and continual improvement, although it is not specific to laboratory activities [10]. In contrast, ISO/IEC 17025 establishes requirements for the competence of testing and calibration laboratories, combining management system requirements with technical criteria to ensure the validity and reliability of laboratory results [7].

ISO 15189 is specifically designed for medical laboratories, integrating quality management principles with technical competence requirements applicable throughout the laboratory workflow, including personnel competence, examination procedures, quality assurance, and risk management [8]. Finally, NIT DICLA 035, issued by Inmetro, establishes the criteria for the recognition of compliance with the Principles of Good Laboratory Practice (GLP) in Brazil, focusing on the organizational and technical requirements necessary to ensure the integrity and reliability of non-clinical laboratory studies [9,11,12]. Although these normative documents differ in scope and structure, they share the common objective of promoting quality, competence, reliability, and continual improvement in laboratory activities.

Despite their different scopes, these standards contain several conceptually similar management and technical requirements. Harmonizing their terminology and identifying equivalent requirements may facilitate the implementation of integrated Quality Management Systems, simplify accreditation processes, and promote greater consistency in laboratory practices [13]. These actions promote reliable results, patient and user safety, and the quality of health services. In addition, this harmonization simplifies the certification or accreditation process, integrating the management, technical, and operational areas between health laboratories [14,15].

Several studies have underscored the importance of quality management in ensuring the reliability of examination data, supporting clinical decision-making, and enhancing patient safety [16,17,18]. Consequently, the implementation of standardized quality control procedures in laboratory facilities directly contributes to improved precision, reproducibility, and consistency in testing, thereby enhancing examination performance and supporting public health strategies [19,20,21].

Standards provide a framework for achieving quality in laboratories by establishing clear, standardized guidelines that address requirements for technical competence, method validation, quality assurance, documentation, and continuous improvement [22]. In this same context, the interpretation of examination results is also subject to sources of technical variability, such as measurement uncertainty, the proper quantification and reporting of which are essential to the reliability of laboratory data [23]. In particular, ISO 15189 has been shown to strengthen quality management systems in clinical laboratories by integrating technical competence with requirements focused on patient needs, thereby adding value to laboratory services and results [24,25]. Although these regulatory documents differ in scope, structure, and terminology, they share several technically and managerially similar requirements. These similarities, together with their structural differences, warrant a comparative analysis to identify areas of convergence, complementarity, and equivalent requirements. By analyzing their conceptual correspondence and terminology, it aims to support the harmonization of normative language, facilitate the integration of QMS and provide greater consistency in the interpretation of quality requirements. While ISO 9001, ISO/IEC 17025, and ISO 15189 are internationally recognized standards, NIT DICLA 035 is a Brazilian regulatory document issued by Inmetro. Accordingly, findings related to this document should be interpreted within the Brazilian regulatory context. Within this context, this article identifies and compares equivalent requirements among four normative documents widely applied in health laboratories aiming to harmonize terminology and support future revisions of quality standards.

## 2. Materials and Methods

### 2.1. Selection of Standards

The standards documents analyzed (ISO 9001:2015, ISO/IEC 17025:2017, ISO 15189:2015, and NIT DICLA 035 (current version)) were selected because they represent complementary frameworks for quality management in health laboratories, encompassing both internationally recognized standards and the Brazilian regulatory context. The ISO standards correspond to the Brazilian adoptions published by ABNT and currently in force in Brazil. Since this study analyzes the requirements of the ISO 15189:2015 standard, the original terminology from that edition (pre-examination, examination, and post-examination) has been retained when referring to the standard. Where appropriate, the text adopts the terminology introduced in ISO 15189:2022 (pre-test, test, and post-test) to ensure greater consistency with current international vocabulary.

### 2.2. Extraction of Requirements

The requirements of each standard were identified through a structured and systematic full-text analysis. Relevant clauses, principles, and mandatory requirements were extracted manually, focusing on those applicable to quality management, technical competence, laboratory processes, data integrity, and regulatory compliance. Only requirements directly related to laboratory practice and quality assurance were included.

### 2.3. Categorization Framework

After extraction, the requirements were categorized into thematic groups to support comparison. The categories were defined based on common elements across standards, including QMS, technical competence, process control, risk management, documentation and records, continuous improvement, result reliability, and validation. This classification enabled a structured comparison across standards with different scopes and purposes.

### 2.4. Comparative Analysis

A cross-comparison matrix was developed to analyze correspondences between the requirements of each standard. The comparison was performed by aligning similar requirements across standards based on their intent, scope, and application in laboratory settings. The analysis prioritized conceptual consistency rather than literal equivalence among texts. The interpretation of conceptual equivalence also took into account internationally accepted metrological terminology, as described in the International Vocabulary of Metrology (IVM), which provides standardized definitions for concepts related to measurement [26].

To ensure consistency and transparency, the relationships between requirements were classified using predefined criteria:

– Equivalence: Requirements that are identical or highly similar in scope, intent, and application.
– Alignment: Requirements that are not identical but are compatible and complementary.
– Same Concept: Requirements addressing the same underlying principle but with different approaches or levels of detail.
– Not Evidenced: Absence of a comparable requirement in the corresponding standard.

A detailed description of classification criteria is provided in Supplementary Table S1.

### 2.5. Data Analysis and Validation

The classification process was conducted iteratively to ensure consistency. Each requirement was reviewed and compared across all standards, ensuring coherence in categorization and minimizing subjective interpretation.

Selection of standards aimed at the implementation of QMS in health laboratories and identification of the main concepts, requirements, and applications of each standard.

i. ISO 9001

ISO 9001:2015 establishes requirements for a QMS applicable to organizations of all types. In health laboratories, it mainly supports management and administrative processes but does not address technical requirements directly, as shown in Supplementary Table S2.

A study published by Gibouleau et al. [27] highlights the successful adaptation of the widely recognized ISO 9001:2015 quality standard in the context of clinical research. The certification of specific activities in clinical research ensures an optimized and relevant organization, focused on the safety of participating volunteers and the scientific quality of research. In addition, this adaptation contributes significantly to the satisfaction of sponsors, who are more willing to invest in research that demonstrates a clear commitment to excellence and safety.

Implementing ISO 9001 in a testing laboratory establishes its organizational culture, allowing activities to be carried out in a more organized way. Through this initiative, systematic guidance is followed to analyze the suitability of its operations [28].

ii. ISO/IEC 17025

The standard ISO/IEC 17025 [7] is specifically designed for testing and calibration laboratories, establishing general requirements for technical competence, impartiality, and consistency of results [29]. Its application ensures the reliability of the data generated in laboratories, which is paramount for testing and calibration laboratories and laboratories that develop systems that have a direct impact on health. Highlights of the importance of ISO/IEC 17025 in laboratories in the health area are presented in Supplementary Table S3.

The ISO/IEC 17025 standard provides a systematic approach to ensure the integrity of samples and the reliability of experimental results. A recently published study discusses the importance of standard laboratory practices in preventing contamination, highlighting that, even with effective precautions, there is a small residual risk of contamination, which can compromise biological specimens and the reliability of laboratory examination results. Thus, laboratories must maintain rigorous testing regimes to monitor and identify high-risk activities. To minimize the risk of cross-contamination, stricter preventive measures based on the requirements of the standard are recommended, such as reorganizing laboratories for “separate” processes [30]. Another analysis addresses the advances and potential of modern screening methodologies. This allows for the identification of many unknown molecules in environmental and biological samples, facilitating the detection of hazardous contaminants and thus helping to prevent health risks. However, for these techniques to be widely adopted and used, it is necessary to implement harmonized quality standards, such as those described in ISO/IEC 17025 [31]. Note that following the implementation of this standard in the management system, ongoing review and improvement are essential in achieving greater laboratory maturity [32].

iii. ISO 15189

ISO 15189 [8] is a standard that establishes specific requirements for technical competence and quality management in clinical laboratories, ensuring the reliability of diagnostic results and the safety of services provided to patients [33]. Unlike other standards in the ISO series, it was developed specifically for laboratories that handle human biological samples, covering aspects such as management, personnel qualification, method validation, and quality control of assays [34]. The standard covers several guidelines and processes, as presented in Supplementary Table S4.

Clinical laboratories process and store sensitive data in most of their activities. In this context, Nikolopoulos et al. [35] highlight the importance of integrating the requirements of the General Data Protection Regulation (GDPR) into medical laboratories operating in accordance with the ISO 15189 standard. These labs handle sensitive data during four main phases: patient arrival and data logging pre-examination, examination, and post-examination, phases. To securely process data, the ISO 15189 standard establishes specific requirements for data management, addressing aspects of security, availability, and protection.

Research also demonstrates the importance of the ISO 15189 standard, which supports the reliability of the activities carried out in clinical laboratories and influences the continuous improvement of laboratory operational processes. This is largely due to the technical requirements set forth by the standard, which include the identification of nonconformities, corrective actions, internal and external quality assessments in clinical laboratories, traceability, and technical accuracy [36,37]. This serves as a reference standard that ensures the reliability of the activities carried out in these laboratories, promoting an integral quality in the services provided [33,38].

iv. NIT DICLA 035

NIT DICLA 035 [9] establishes the requirements that recognize compliance with the principles of GLP, promoting the international acceptance of studies conducted in Brazil through alignment with the guidelines of the Organization for Economic Co-operation and Development (OECD). This standard is mainly applied to non-clinical studies related to health and safety, covering organizational and operational aspects that ensure the quality, traceability, and reliability of the data generated [39]. Some highlights of this standard that can be applied to laboratories in the health area are presented in Supplementary Table S5.

GUAN et al. [40] deal with the importance of pipettes as essential tools in biomedical and testing laboratories, from the perspective of GLP. In this review, manual pipetting is highlighted as a fundamental technique in the laboratory; however, despite its apparent simplicity, it is subject to imprecision and low accuracy. The authors emphasize that having a common protocol for evaluating variations in procedures enables cross-laboratory comparisons and can facilitate the establishment of reference values for acceptable ranges of operator error [40]. In this sense, implementing GLP ensures that laboratories adhere to and promote the best practices and quality standards. This meets the demand for a broad training program, involving all spheres of management, that promotes a culture of quality and safety in public institutions and better prepares students for the job market [41].

## 3. Results and Discussion

### 3.1. Quality Management in Health Laboratories

Quality management in laboratories has evolved significantly over the past few decades, especially with the advancement of international norms and standards; the development of automation and information technologies; and the increasing emphasis on quality assurance and safety [42]. In this sense, studies prove that having mechanisms that guarantee a normative approach to quality in health laboratories ensures the integrity of information, which is fundamental for clinical decision-making, the development of public health policies, and the advancement of scientific research. This ultimately ensures that the results are valid and reproducible [43]. In addition, studies reveal that health information is fundamental for the planning, programming, monitoring, and management of individual and collective health interventions, enabling the monitoring of health goals [44].

Generally, quality in laboratories can be characterized by the adoption of international and national standards that establish guidelines and requirements to ensure operational excellence and the reliability of results [45,46,47]. These standards must be adapted to the organizational context through the implementation of policies, processes, and procedures that meet both management and technical requirements. Depending on their scope, they may apply exclusively to management systems or extend to technical and operational laboratory activities. These can be applied solely to managing or, depending on the standard, extended to cover and ensure this scope of quality to technical and operational activities.

Although this study focuses on the ISO-based framework adopted in Brazil, it is important to recognize that laboratory quality management is not governed by a single international model. In North America, for example, medical laboratories are supported by a broader set of organizations and guidance documents, including the College of American Pathologists (CAP) and the Clinical and Laboratory Standards Institute (CLSI) in the United States, as well as the Canadian Society for Medical Laboratory Science (CSMLS), the Institute for Quality Management in Healthcare (IQMH), and Accreditation Canada in Canada. Although these organizations adopt different regulatory approaches, they share the common objective of promoting laboratory quality, technical competence, and patient safety [48]. QMSs must be implemented to ensure quality requirements in health laboratories, covering all stages of the laboratory process, from data collection to the preparation of technical reports. There is a broad consensus on the importance of laboratories adopting a QMS to ensure the quality of their work, acknowledging, however, the peculiar difficulties faced by research and development (R&D) laboratories in the implementation of such systems. The definition of specific requirements based on ISO/IEC 17025 standards is an important step in adapting the QMS to the needs of research laboratories. Thus, an agile methodology is proposed to meet these needs by offering a flexible and adaptable approach, facilitating the implementation and continuous operation of the QMS in R&D environments.

Standards have specific peculiarities and scopes, which means that each set of standards has its own characteristics and areas of application. In health laboratories, which operate in different areas, this diversity is even more pronounced. Therefore, in the implementation of QMS, one must consider both the applicable standards and the variety of activities performed. Thus, the definition of specific requirements based on ISO/IEC 17025 standards can support this context, as this internationally recognized standard establishes strict criteria for technical competence and quality management in laboratories. Adapting the QMS according to these requirements ensures that processes meet standards and are appropriate for the specific needs of R&D laboratories. Adopting an agile and adaptable approach facilitates not only the initial implementation of the QMS but also its continued operation in R&D environments, which are often dynamic and subject to rapid change. This type of methodology allows for quick and efficient adjustments, ensuring that the QMS remains effective and relevant even in the face of new demands and challenges [49]. In this sense, standards that can contemplate all phases and activities of a laboratory should be applied, including areas of support.

### 3.2. Comparative Analysis of Concepts Related to Requirements and Their Applications: Systemic View of ISO 9001, ISO/IEC 17025, ISO 15189, and NIT DICLA 035 Standards

To present a comprehensive and comparative view of the standards discussed in this study, an exploratory analysis of the concepts was carried out, mainly related to the requirements and their applications. Table 1 presents a comparison of the quality management standards considered in this study.

### 3.3. Equivalence and Terminological Harmonization of Requirements

Due to the disparities between groups that elaborate and structure the standards here described, ISO in contrast to Inmetro’s GLP standard, the resulting lack of uniformity makes it challenging to evaluate them comparatively in terms of requirements. These distinctions can be observed in requirements that apply only in specific contexts, both in relation to the processes and procedures of the technical area and the management area. In addition, it is important to highlight that the comparison is difficult due to the specific language and terms adopted by NIT DICLA 035, whose regulatory nature makes its application mandatory, even in specific situations.

The ISO 15189 standard establishes a normative framework that divides the requirements into “management” and “technical” groups, facilitating its application throughout the laboratory, regardless of scope. Conversely, ISO/IEC 17025 and NIT DICLA 035 approach requirements as a sequence in the process or study, adopting them as a checklist to be followed, with a defined scope for specific tests, calibrations, or studies, even if a cross-cutting QMS is implemented.

Notably, ISO 9001 introduces the perspectives of “process performance” and sustainability, highlighting the importance of understanding and managing an organization’s activities as a set of related processes, rather than isolated functions. However, it is evident that this standard is not about issues related to safety or the scientific quality of research; rather, it certifies the system, disregarding the technical specificities.

**Figure 1:**
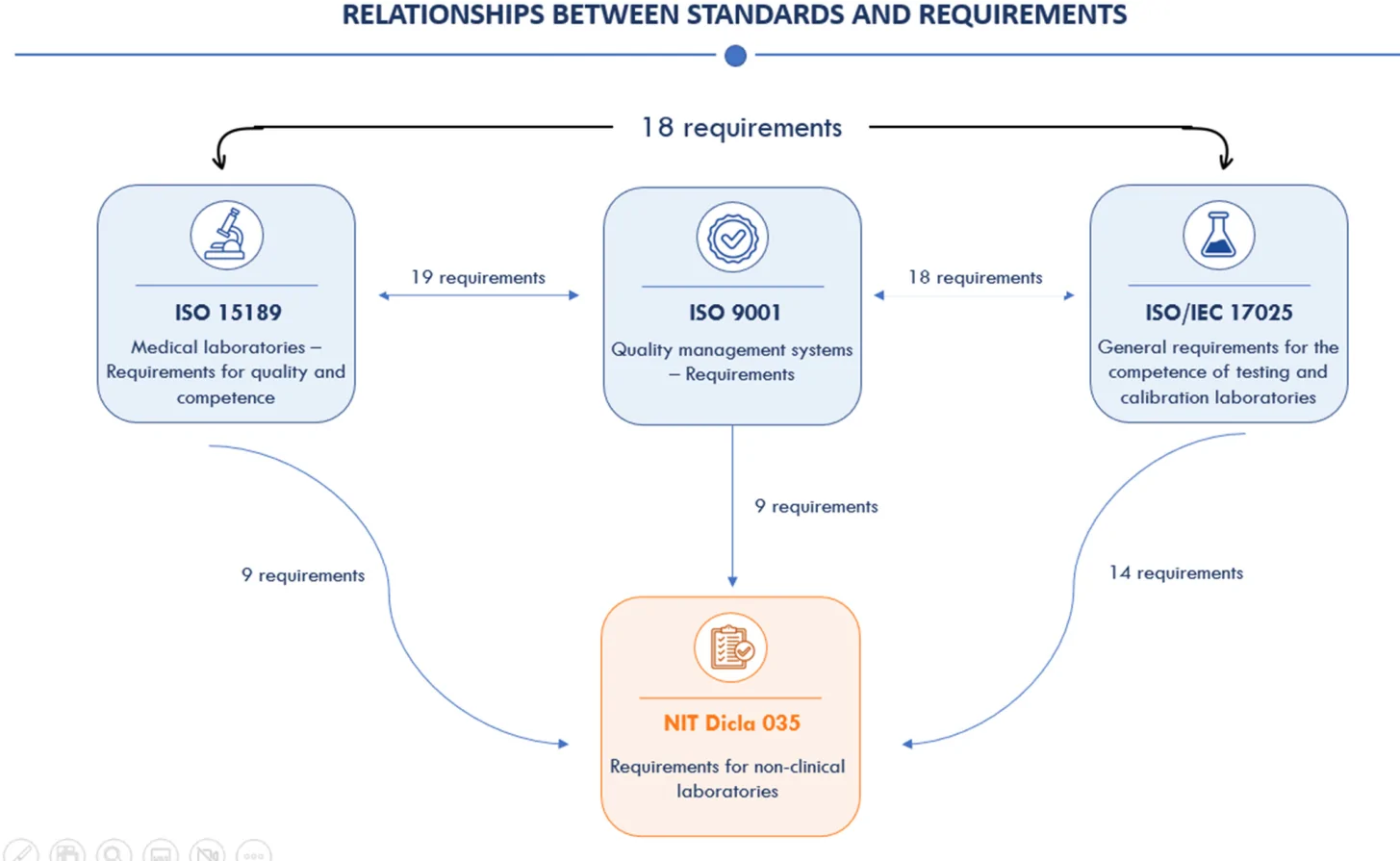
Interrelationship between the requirements among the standards evaluated. The comparison presented in Figure 1 demonstrates that the greatest conceptual convergence occurs among ISO 9001, ISO/IEC 17025, and ISO 15189, particularly regarding quality management principles, document control, corrective actions, internal audits, and personnel competence. These requirements represent the core elements of laboratory Quality Management Systems and constitute the basis for harmonized implementation across different laboratory contexts. In contrast, NIT DICLA 035 presents fewer direct correspondences because of its regulatory structure and its focus on Good Laboratory Practice, although it shares several management-related requirements with the ISO standards. These findings reinforce that the main differences among the standards are associated with their scope and terminology rather than with their underlying quality management principles.

In a more detailed view, the ISO 9001 standard has 24 requirements: 9 are in common with the other standards, 19 are correlated with ISO 15189, 18 are correlated with ISO/IEC 17025, and 9 are correlated with NIT DICLA 035. In general, ISO 9001 is incorporated into all of the quality management standards mentioned here. Figure 1 illustrates the interrelationships and conceptual similarity between the various quality requirements of the four normative documents examined in this study. Although two of the standards are based on ISO 9001, each has its own peculiarities, formats, and specific concepts, namely ISO 17025 and ISO 15189. In addition, NIT DICLA 035, a GLP-based regulation, also contains several management requirements that are equivalent to those of ISO 9001; however, they are not grouped, and the technical requirements do not follow the same format.

Implementing ISO 9001, ISO 17025, ISO 15189, and NIT DICLA 035 in healthcare laboratories faces a few challenges. First, these standards require a significant change in organizational culture, as they require an ongoing commitment to quality—from senior management to operational support [50]. In addition, adapting existing processes to meet standard requirements can be complex and time-consuming, especially in laboratories where traditional practices may be entrenched. Achieving and maintaining requirements for compliance with these standards also requires investments in staff training, infrastructure, and technology, which can pose a financial challenge for many laboratories, particularly those with limited resources [51,52,53].

To ensure the accuracy and reliability of test reports and laboratory results, researchers have dedicated themselves to examining laboratories that handle biological samples [54]. One of the main paths adopted is to obtain accreditation or certification, presenting a lab as a guarantee of quality and compliance with internationally recognized norms or standards. This accreditation process not only reinforces confidence in the results obtained by laboratories but also ensures that their practices are aligned with the best international standards, contributing to excellence and competitiveness in the healthcare field. In this way, achieving accreditation and certification becomes a step in continuous improvement, thus maintaining confidence in services provided by health laboratories [18].

Understanding the importance of a well-implemented Quality Management System in research environments and health services requires a rigorous methodological approach. For example, the adoption of Good Laboratory Practice guidelines has ensured the quality and integrity of services in the United States, directly benefiting public health and scientific research [55].

#### 3.3.1. Detailed Analysis Based on ISO 9001

**Figure 2:**
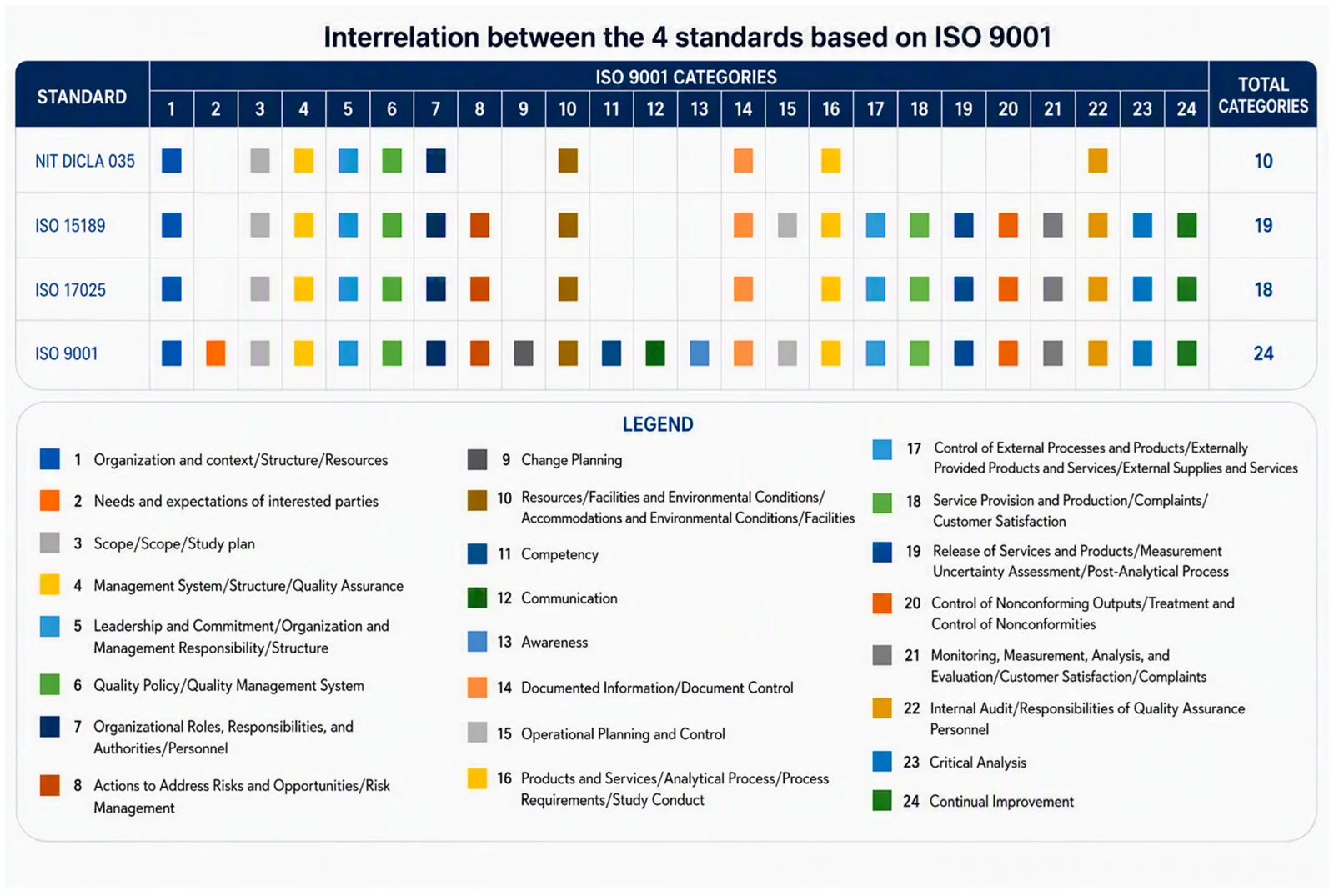
Interrelationships between standards based on ISO 9001.

The ISO 9001 standard was used as a reference framework because of its broad applicability in QMS across different sectors. Among its 24 requirements, 18 showed conceptual alignment with the ISO/IEC 17025 and ISO 15189 standards, while 10 showed equivalence across all four normative documents. It is important to note that, despite differences in terminology, similar requirements could be identified across the standards. Six requirements—“Needs and expectations of interested parties,” “Planning of changes,” “Competence,” “Communication,” “Awareness,” and “Operational planning and control”—did not have an explicit correspondence in the other standards. Table 2 presents a detailed classification according of the degree of correspondence and Figure 2 summarizes these interrelationships among the standards.

Overall, the analysis highlights a high level of convergence among the standards, despite differences in terminology and in the level of technical detail. These findings indicate thar harmonizing terminology and mapping equivalent requirement can facilitate the integration of Quality Management Systems, simplify compliance with multiple standards, and promote a more consistent and efficient approach to quality management in health laboratories.

#### 3.3.2. Analysis Based on ISO 15189

**Figure 3:**
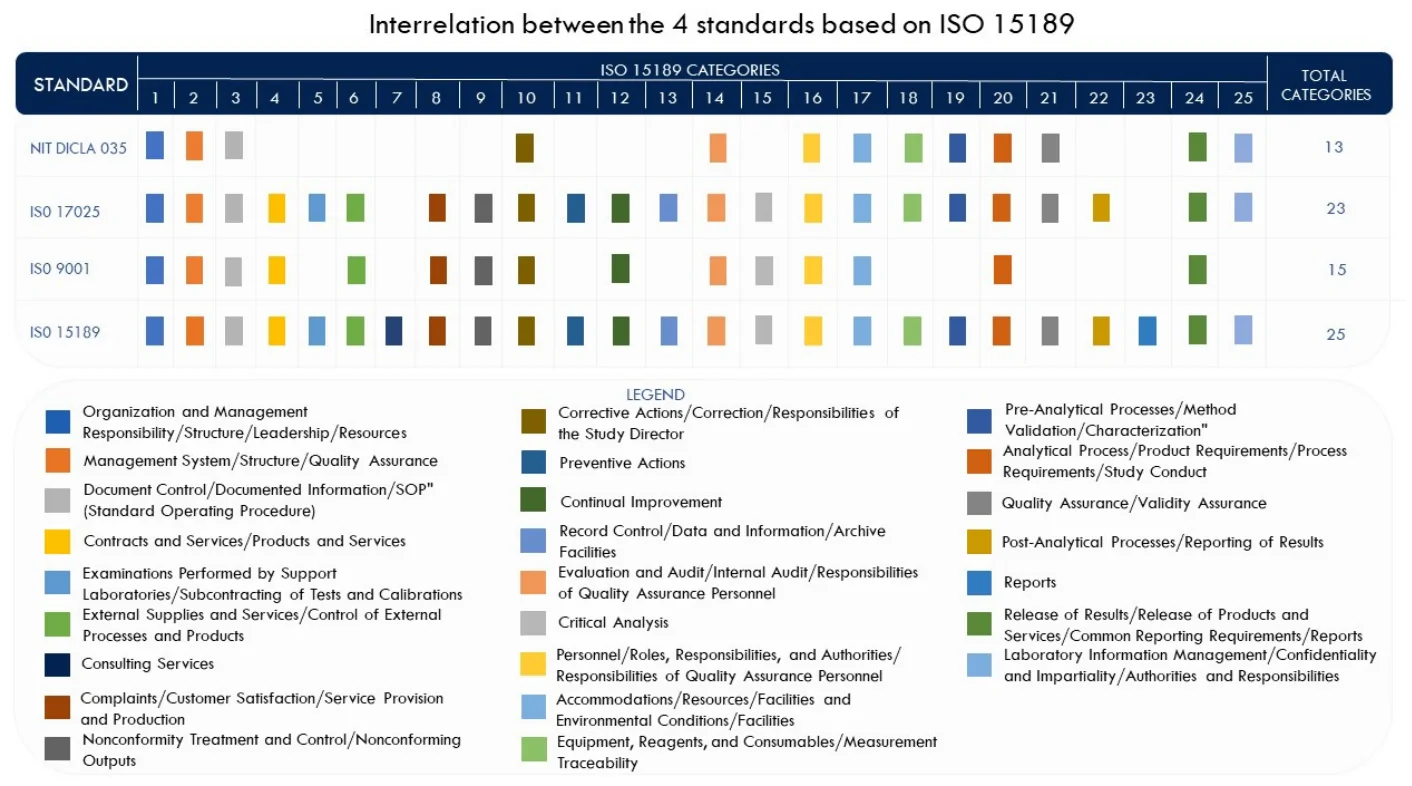
Interrelationship between standards based on ISO 15189.

The 25 requirements of ISO 15189 were used as a reference framework due to their specific focus on clinical laboratories, technical competence, and regulatory compliance in healthcare services. Figure 3 summarizes the conceptual relationships between the evaluated standards, while Table 3 presents a detailed classification of the degree of correspondence [8,52].

Comparison of the four normative documents using ISO 15189 as the reference showed that 9 of the 25 requirements were equivalent across all four standards. In contrast, the requirements “Consulting services” and “Reports” are found exclusively in ISO 15189. Another noteworthy finding concerns the requirements for “Equipment, reagents, and consumables,” “Pre-examination process,” “Quality assurance,” and “Laboratory information management,” none of which are identified in ISO 9001. The latter item addresses the management of conflicts of interest, the protection of data, and the maintenance of the laboratory’s independence from all interested parties.

When comparing the 25 requirements listed in ISO 15189 with standards from the same family (ISO 9001 and ISO/IEC 17025), 14 common requirements were identified: “Organization and management responsibility,” “Management system,” “Contracts and services,” “External supplies and services,” “Complaints,” “Handling and control of non-conformities,” “Continuous improvement,” “Evaluation and audit,” “Management review,” “Personnel,” “Facilities,” “examination process,” “Release of results,” and “Complaints.” Evidence of greater alignment between ISO/IEC 17025 and ISO 15189 revealed four requirements found only in these two approaches. These discrepancies highlight the need for terminology harmonization, given that differences in requirement nomenclature may not result in direct equivalence regarding implementation, impact, and criticality. Therefore, standardizing these requirements is essential to ensure the uniform and efficient application of standards, thereby fostering reliability in laboratory health services.

Comparison based on ISO 15189 revealed a higher level of equivalence than analysis based on ISO 9001, indicating that ISO 15189 is structurally closer to ISO/IEC 17025, as both standards were developed specifically for laboratory activities. Conversely, ISO 9001 provides a broader management framework applicable to organizations in general and, consequently, includes requirements not explicitly addressed in laboratory-specific standards.

Although most requirements were conceptually equivalent, differences in terminology and level of detail remain significant challenges for harmonization. These variations do not necessarily represent conflicting requirements but can influence interpretation, implementation, and auditing processes. Standardizing terminology and improving cross-references between standards would facilitate the integration of QMS and reduce the duplication of effort in laboratories operating under multiple regulatory frameworks [56]. These findings corroborate the feasibility of integrating QMS across different normative documents while emphasizing that successful implementation depends on the careful interpretation of terminology and consideration of the technical specifics of each laboratory environment.

## 4. Conclusions

This comparative analysis of ISO 9001, ISO/IEC 17025, ISO 15189, and NIT DICLA 035 demonstrates a substantial level of convergence among quality management standards applied to health laboratories. The results showed that, depending on the analytical perspective, requirements may present different levels of correspondence, ranging from alignment to full equivalence. Notably, when ISO 15189 was used as the reference, equivalent requirements were predominantly observed, indicating a strong structural compatibility with ISO/IEC 17025 and, to a lesser extent, ISO 9001.

These findings confirm that implementing integrated Quality Management Systems across different standards is technically feasible, although this requires careful interpretation of terminology and attention to differences in the level of detail and technical specificity. In particular, ISO 9001 provides a broader managerial framework, while ISO 15189 and ISO/IEC 17025 incorporate more specialized technical requirements for laboratory environments.

Despite the overall convergence, variations in terminology and the explicitness of certain requirements highlight ongoing challenges for full harmonization. Addressing these differences through improved standardization of terminology and clearer cross-referencing between standards could facilitate integration and improve consistency in implementation and auditing processes.

This study has some limitations that should be acknowledged. First, the analysis was restricted to four normative documents commonly applied in health laboratories and did not include other internationally relevant regulatory and accreditation frameworks, such as the Clinical Laboratory Improvement Amendments (CLIA) and the College of American Pathologists (CAP), which are widely used in the United States, or other reference organizations, including the Clinical and Laboratory Standards Institute (CLSI) and the Canadian Society for Medical Laboratory Science (CSMLS). Consequently, the findings cannot be directly generalized to regulatory environments outside the ISO/ABNT/Inmetro framework. Second, the study was based exclusively on document analysis and did not evaluate the practical implementation of these standards in real laboratory settings. Nevertheless, the results provide a robust basis for understanding the relationships among quality standards and may support future efforts toward integrating quality management systems and expanding comparative analyses across different international regulatory frameworks.

## Figures and Tables

**Table 1:** Comparison of quality standards in healthcare laboratories: concepts and applications.

Standard	ISO 9001 [6]	ISO/IEC 17025 [7]	ISO 15189 [8]	NIT DICLA 035 [9]
Scope	Covers a wide variety of organizations, regardless of the type of product or service	Applied in laboratories that perform calibration and testing	Specifically designed for medical/clinical and healthcare laboratories	Adopted for environmental and health safety studies required by regulatory agencies for registration of some products
Main Focus	Overall quality management and continuous improvement across organizational processes	Technical competence of the laboratory, covering calibration and testing processes	Laboratory activities, ensuring technical competence and the management system in clinical laboratories	Organizational processes and their conditions assess the environmental risks and human health of those involved in the study
Goal	Implements continuous improvement for customer satisfaction and system, showing effectiveness in quality management	Ensures technical competence and reliability of results in calibration and testing laboratories	Ensures that clinical laboratory services meet the standards of quality, accuracy, and reliability required for healthcare	Establishes procedures and documents used in the recognition of compliance with GLP principles
Requirements	Generic; applicable to a variety of organizations, including requirements related to leadership, planning, support, operation, performance, and evaluation	Specific to calibration and testing laboratories, including impartiality and confidentiality	Specific to clinical laboratories, covering technical and management requirements	Specific for registration or licensing of pharmaceuticals, pesticides, cosmetics, veterinarians, food and feed additives, and industrial chemicals
Staff	Focuses on the overall competence of staff in relation to roles and responsibilities	Emphasizes the determination of competency, selection, training, supervision, and authorization requirements of personnel	It has specific requirements regarding the training and assessment of technical competence of laboratory personnel	Defines responsibilities at all levels; emphasizes qualification and experience to supervise all phases of studies
Documents	Addresses document and record control in general	Procedures for controlling documents and technical records	Highlights the importance of documented information and specific records for laboratory activities	Requires secure storage and retrieval of all study plan information
Internal Audit/Inspections	Verifies compliance with standard requirements	Ensures the quality of laboratory processes	Ensures technical competence of the laboratory	Guarantees that the laboratory follows the standards established for carrying out regulated studies
Continuous Improvement	Actions needed to meet requirements and increase stakeholder satisfaction	Must identify and select opportunities for improvement	For the effectiveness of the Quality Management Systems, including critical analysis and performance	Not evidenced
Infrastructure Facilities Environment	Determines the maintenance required for process operation and compliance of products and services	Emphasizes control when there is influence on results	Considers process security, including access control and security devices	Facilitates the development of the study, including biosafety and the environment
Quality Management	Specific requirement to establish, implement, and maintain a QMS	Specific requirement to establish, document, and maintain policies	Specific requirements and must provide integration of all processes	Generic requirement for performance and verification of GLP compliance and approval of the study plan
Samples	Not evidenced	Specific requirement to have sampling plans and methods, including selection and treatment	Specific requirement for receiving transport, handling, preparation, and storage	Specific requirement for receiving handling, sampling, and storage
Method Validation	Not evidenced	Use of validated examination methods; evaluates measurement uncertainties and statistical techniques	Emphasizes the use of validated clinical examination methods	Reference to OECD Test Guides and other recognized methods
Traceability	Controls unique identification and retains documented information	Metrologic: Establishes and maintains a calibration program	Requires controls of metrological process records for analyses	Traceability for study reconstruction
Quality Control	Process and product control	Internal controls to monitor accuracy and consistency; external process and testing controls	Internal quality controls in each examination	Inspection and approval of the study plan
Equipment Calibration and Maintenance	Not evidenced	Ability to achieve measurement accuracy or uncertainty to provide valid results	Process for selection, procurement, and control of equipment	Retention of equipment maintenance and calibration records
Reviews	Regular reviews of the management system.	Reviews by management to ensure continued effectiveness	Revisions by management, including technical revision	Review by the Study Director, from execution to the final report of each study
Non-Conformities	Specific requirement to ensure compliance with quality requirements	Specific requirement defining responsibilities and authorities for resolution	Specific requirement to identify and address non-conformities	Not evidenced
Preventive Action	Not specified	Generic requirement to ensure compliance with quality requirements	Specific requirement to eliminate causes of potential non-conformities	Not evidenced
Complaints/Customer Satisfaction	Specific customer-focus requirement	Specific requirement for handling complaints	Specific requirement for handling complaints by keeping records of actions taken	Not evidenced

**Table 2:** Requirements defined by ISO 9001 and correlated with ISO/IEC 17025, ISO 15189, and NIT DICLA 035 based on the degree of requirement correspondence.

ISO 9001 Requirements	IS0 9001	IS0 17025	IS0 15189	NIT DICLA035
4.1 Context of organization	Equivalence	Alignment	Alignment	Alignment
4.2 Interested parties	Equivalence	Not evidenced	Alignment	Not evidenced
4.3 Scope	Equivalence	Alignment	Alignment	Alignment
4.4 QMS	Equivalence	Equivalence	Equivalence	Equivalence
5.1 Leadership	Equivalence	Alignment	Alignment	Alignment
5.2 Quality policy	Equivalence	Equivalence	Equivalence	Alignment
5.3 Roles and responsibilities	Equivalence	Equivalence	Equivalence	Equivalence
6.1 Risk and opportunities	Equivalence	Alignment	Alignment	Alignment
6.3 Change planning	Equivalence	Not evidenced	Not evidenced	Not evidenced
7.1 Resources	Equivalence	Equivalence	Equivalence	Equivalence
7.2 Competence	Equivalence	Same concept	Same concept	Same concept
7.3 Awareness	Equivalence	Alignment	Alignment	Alignment
7.4 Communication	Equivalence	Alignment	Alignment	Alignment
7.5 Documented information	Equivalence	Equivalence	Equivalence	Equivalence

**Table 3:** Requirements defined by ISO 15189 and correlated with ISO 9001, ISO/IEC 17025, and NIT DICLA 035 based on the degree of requirement correspondence.

ISO 15189 Requirements	ISO 15189	IS0 9001	ISO 17025	NIT DICLA 035
4.1 Organization	Equivalence	Alignment	Equivalence	Equivalence
4.2 Management system	Equivalence	Equivalence	Equivalence	Equivalence
4.3 Document control	Equivalence	Equivalence	Equivalence	Equivalence
4.4 Service agreements	Equivalence	Alignment	Alignment	Alignment
4.5 Referral labs	Equivalence	Not evidenced	Alignment	Alignment
4.6 External services	Equivalence	Equivalence	Equivalence	Equivalence
4.7 Consulting	Equivalence	Not evidenced	Not evidenced	Not evidenced
4.8 Complaints	Equivalence	Alignment	Alignment	Alignment
4.9 Nonconformities	Equivalence	Equivalence	Equivalence	Equivalence
4.10 Corrective actions	Equivalence	Equivalence	Equivalence	Equivalence
4.11 Preventive actions	Equivalence	Same concept	Alignment	Alignment
4.12 Continuous improvement	Equivalence	Same concept	Same concept	Equivalence
4.13 Records	Equivalence	Alignment	Equivalence	Equivalence
4.14 Audits	Equivalence	Equivalence	Equivalence	Equivalence
4.15 Management review	Equivalence	Same concept	Same concept	Equivalence
5.1 Personnel	Equivalence	Same concept	Equivalence	Equivalence
5.2 Facilities	Equivalence	Alignment	Equivalence	Equivalence
5.3 Equipment	Equivalence	Not evidenced	Equivalence	Equivalence
5.4 Pre-examination	Equivalence	Not evidenced	Equivalence	Equivalence
5.5 Examination	Equivalence	Alignment	Equivalence	Equivalence
5.6 Quality assurance	Equivalence	Not evidenced	Equivalence	Equivalence
5.7 Post-examination	Equivalence	Not evidenced	Alignment	Alignment
5.8 Reporting	Equivalence	Alignment	Equivalence	Equivalence
5.9 Result release	Equivalence	Alignment	Equivalence	Equivalence
5.10 Information management	Equivalence	Not evidenced	Alignment	Equivalence

## Data Availability

The original contributions presented in this study are included in the article/Supplementary Material. Further inquiries can be directed to the corresponding author.

## Supplementary Materials

The following supporting information can be downloaded at: https://www.mdpi.com/article/10.3390/biotech15030069/s1, Table S1. Definition of the terms used in comparative analyses of standards; Table S2. Key elements and description of principles of ISO 9001; Table S3. Key elements and description of principles of ISO/IEC 17025; Table S4. Key elements and description of the principles of ISO 15189; Table S5. Key elements and description of principles of NIT DICLA 035.

## Abbreviations

The following abbreviations are used in this manuscript:

ABNT	Brazilian Association of Technical Standards
CONMETRO	National Council of Metrology, Standardization and Industrial Quality
GDPR	General Data Protection Regulation
GLP	Good Laboratory Practice
IEC	International Electrotechnical Commission
INMETRO	National Institute of Metrology, Quality and Technology
ISO	International Organization for Standardization
OECD	Organization for Economic Cooperation and Development
QMS	Quality Management System
R&D	Research and Development
Sinmetro	National System of Metrology, Standardization and Industrial Quality

## References

[1] Kinyenje E., Ngowi R.R., Msigwa Y.S., Hokororo J.C., Yahya T.A., German C.J., Mawazo A., Mohamed M.A., Nassoro O.A., Degeh M.M. (2023). Status of countrywide laboratory services quality and capacity in primary healthcare facilities in Tanzania: Findings from Star Rating Assessment. PLoS Glob. Public Health.

[2] Plebani M., Sciacovelli L., Chiozza M.L., Panteghini M. (2015). Once upon a time: A tale of ISO 15189 accreditation. Clin. Chem. Lab Med..

[3] Ohn H.M. (2024). Internal audit techniques for testing laboratories: ISO/IEC 17025:2017 perspective. Accredit. Qual. Assur..

[4] Lebedynets V., Tkachenko O. (2017). Formation of the quality management systems at state laboratories for a medicines control. Technol. Transf. Innov. Solut. Med..

[5] Kariuk A. (2017). Technology Transfer: Innovative Solutions. Fundamental and Applied Physics.

[6] (2015). Quality and Competence Management System.

[7] (2017). General Requirements for the Competence of Testing and Calibration Laboratories.

[8] (2015). Medical Laboratories—Requirements for Quality and Competence.

[9] Instituto Nacional de Metrologia, Qualidade e Tecnologia (Inmetro) (2019). NIT-DICLA-035 Principles of Good Laboratory Practice.

[10] Idris M.C., Durmuşoğlu A. (2023). An empirical examination of ISO 9001′s influence on sustained success of companies. Accredit. Qual. Assur..

[11] Tsimillis K.C. (2019). Data integrity: Requirements set in the accreditation standards. Accredit. Qual. Assur..

[12] Instituto Nacional de Metrologia, Qualidade e Tecnologia (Inmetro) (2011). NIT-DICLA-035—Principles of Good Laboratory Practice—BPL.

[13] Hasdiana U. (2018). Laboratory Quality Standards and their Implementation. Anal. Biochem..

[14] Buchta C., Huf W., Badrick T. (2025). Cascading referencing of terms and definitions. Clin. Chem. Lab Med..

[15] Nordin G., Dybkaer R., Forsum U., Fuentes-Arderiu X., Pontet F. (2018). Vocabulary on nominal property, examination, and related concepts for clinical laboratory sciences (IFCC-IUPAC Recommendations 2017). Pure Appl. Chem..

[16] Rusanganwa V., Nzabahimana I., Evander M. (2024). Quality and resilience of clinical laboratories in Rwanda: A need for sustainable strategies. Glob. Health Action.

[17] Olver P., Bohn M.K., Adeli K. (2023). Central role of laboratory medicine in public health and patient care. Clin. Chem. Lab. Med..

[18] Jang M.A., Yoon Y.A., Song J., Kim J.H., Min W.K., Lee J.S., Lee Y.W., Lee Y.K. (2017). Effect of Accreditation on Accuracy of Diagnostic Tests in Medical Laboratories. Ann. Lab Med..

[19] Dong P., Wang Y.B., Peng D.Z., Wang J.J., Cheng Y.T., Deng X.Y., Zheng B., Tao R. (2021). Utility of process capability indices in assessment of quality control processes at a clinical laboratory chain. J. Clin. Lab Anal..

[20] Pereira P. (2024). Trends in the Accreditation of Medical Laboratories by ISO 15189. Six Sigma and Quality Management.

[21] Lane R. (2021). Pascale Ondoa: Strengthening laboratory medicine in Africa. Lancet.

[22] Pum J. (2019). A practical guide to validation and verification of analytical methods in the clinical laboratory. Adv. Clin. Chem..

[23] Possolo A., Hibbert D., Stohner J., Bodnar O., Meija J. (2024). A brief guide to measurement uncertainty (IUPAC Technical Report). Pure Appl. Chem..

[24] Plebani M., Sciacovelli L. (2017). ISO 15189 Accreditation: Navigation Between Quality Management and Patient Safety. J. Med. Biochem..

[25] Cubukcu H.C., Vanstapel F., Thelen M., Bernabeu-Andreu F.A., van Schrojenstein Lantman M., Brugnoni D., Brguljan P.M., Milinkovic N., Linko S., Vaubourdolle M. (2021). Improving the laboratory result release process in the light of ISO 15189:2012 standard. Clin. Chim. Acta.

[26] Joint Committee for Guides in Metrology (JCGM) (2012). International Vocabulary of Metrology—Basic and General Concepts and Associated Terms (VIM), JCGM 200:2012 (ISO/IEC Guide 99:2007).

[27] Gibouleau C., Padioleau C., Poinas A., Vrignaud F., Deblois S., Galisson I.M., Lecerf P., Fuzeau C., Dreno B., Trochu J.N. (2019). Réussir une démarche de certification ISO 9001 v 2015 en investigation clinique. Therapies.

[28] Escobedo Rosales L.G., Treviño Uribe J.J., Alcalá Salinas C.A., Zapata Rebolloso A. (2023). Implementación de ISO 9001:2015 en un Laboratorio de Ensayo. Cienc. Lat. Rev. Multidiscip..

[29] Miguel A., Moreira R., Oliveira A. (2021). ISO/IEC 17025: History and introduction of concepts. Quim. Nova.

[30] Henry E., Charalambous E., Betsou F., Mathieson W. (2024). Implementing routine monitoring for nuclease contamination of equipment and consumables into the quality management system of a laboratory. Heliyon.

[31] Monteiro Bastos da Silva J., Chaker J., Martail A., Costa Moreira J., David A., Le Bot B. (2021). Improving Exposure Assessment Using Non-Targeted and Suspect Screening: The ISO/IEC 17025: 2017 Quality Standard as a Guideline. J. Xenobiot..

[32] de Jesus L.N., Penteado R.B., Malheiros F.C., Medrano Castillo L.A., de Almeida L.F.M. (2023). The conception and initial years of a quality management system based on ISO/IEC 17025: An action research. Accredit. Qual. Assur..

[33] Azua-Menéndez M., Bravo-Chichande V.P., Chancay-Pincay G.J. (2024). Importancia de la aplicación de las normas ISO 15189 en los laboratorios clínicos. MQRInvestigar.

[34] Silva M.M. (2023). Implementation of quality management in a clinical laboratory: Experience report. J. Med. Health Promot..

[35] Nikolopoulos M., Karampela I., Antonakos G., Tzortzis E., Stratigou T., Diomidous M., Dalamaga M. (2019). Integrating GDPR in ISO 15189 for Medical Laboratories: Major Aspects and Perspectives. Stud. Health Technol. Inform..

[36] Yang S., Zhou Y., Wang C., Luo M. (2026). The “Double Helix” model of quality monitoring: Risk mapping of quality management system during initial ISO 15189 Implementation in a medical laboratory. PLoS ONE.

[37] Gruber L., Hausch A., Mueller T. (2024). Internal Quality Controls in the Medical Laboratory: A Narrative Review of the Basic Principles of an Appropriate Quality Control Plan. Diagnostics.

[38] Chandra S., Kusum A., Gaur D., Chandra H. (2022). Analytical and post analytical phase of an ISO 15189:2012 Certified cytopathology laboratory-a five year institutional experience. J. Cytol..

[39] OECD O for EC and Development (2023). OECD Council Recommendations. https://www.oecd.org/env/tools-evaluation/oecdcouncilrecommendations.html.

[40] Guan X.L., Chang D.P.S., Mok Z.X., Lee B. (2023). Assessing variations in manual pipetting: An under-investigated requirement of good laboratory practice. J. Mass Spectrom. Adv. Clin. Lab..

[41] Furtado T.T., Machado L.A.R. (2022). Fundamentals of Good Laboratory Practice. Extensão Tecnológica Ext. J. Fed. Inst. St. Catarina (IFC).

[42] Sciacovelli L., Padoan A., Aita A., Basso D., Plebani M. (2023). Quality indicators in laboratory medicine: State-of-the-art, quality specifications and future strategies. Clin. Chem. Lab Med..

[43] Samsa, Greg L.S. (2019). A Guide to Reproducibility in Preclinical Research. Acad. Med..

[44] Malta D.C., Szwarcwald C.L. (2017). Population-based surveys and monitoring of noncommunicable diseases. Rev. Saude Publica.

[45] Fritzer-Szekeres M. (2010). Quality management in medical laboratories. Hamostaseologie.

[46] Marsden A., Shahtout A. (2024). International Organization for Standardization. Clinical Laboratory Management.

[47] Sciacovelli L., Lippi G., Sumarac Z., West J., del Pino Castro I.G., Vieira K.F., Ivanov A., Plebani M. (2017). Quality Indicators in Laboratory Medicine: The status of the progress of IFCC Working Group “Laboratory Errors and Patient Safety” project. Clin. Chem. Lab Med..

[48] Canadian Society for Medical Laboratory Science (CSMLS) (2024). The Use of Standards in the Medical Laboratory: Position Statement. https://csmls.org/wp-content/uploads/2025/03/PS_EN-The-Use-of-Standards-in-the-Medical-Laboratory.pdf.

[49] Martínez-Perales S., Ortiz-Marcos I., Ruiz J.J. (2021). A proposal of model for a quality management system in research testing laboratories. Accredit. Qual. Assur..

[50] Wolniak R., Olkiewicz M. (2019). The Relations Between Safety Culture and Quality Culture. Syst. Saf. Hum. Tech. Facil. Environ..

[51] Okezue M.A., Adeyeye M.C., Byrn S.J., Abiola V.O., Clase K.L. (2020). Impact of ISO/IEC 17025 laboratory accreditation in sub-Saharan Africa: A case study. BMC Health Serv. Res..

[52] Leite L.G.F., de Senna Junior V.A. (2023). Quality and Competence of Clinical Laboratories Regarding ISO 9001 and ISO 15189. REASE.

[53] Ong S.K., Donovan G.T., Ndefru N., Song S., Leang C., Sek S., Noble M., Perrone L.A. (2020). Strengthening the clinical laboratory workforce in Cambodia: A case study of a mixed-method in-service training program to improve laboratory quality management system oversight. Hum. Resour. Health.

[54] Sack U., Bossuyt X., Andreeva H., Antal-Szalmás P., Bizzaro N., Bogdanos D., Borzova E., Conrad K., Dragon-Durey M.A., Eriksson C. (2020). Quality and best practice in medical laboratories: Specific requests for autoimmunity testing. Autoimmun. Highlights.

[55] de Souza R.A., Docena C., dos Santos Silva P., da Silva A.B.M., Brum A.P.O. (2012). Implementation of Good Laboratory Practices (NIT-DICLA-035, Inmetro) in a technological platforms network: The Fiocruz experience. Accredit. Qual. Assur..

[56] Pradella M. (2023). ISO guidelines for laboratory developed tests, emerging technologies, validation and verification—Indicazioni ISO per esami sviluppati in laboratorio, tecnologie emergenti, validazione e verifica. Indicazioni ISO per Esami Sviluppati in Laboratorio, Tecnologie Emergenti, Validazione e Verifica.

