# Supplementary material for: Analysis of Quality Normative Requirements: Contributions to the Alignment and Standardization of Standards Applied in Health Laboratories
Source: BioTech (Basel). 2026 Aug 19;15(3):69. doi: 10.3390/biotech15030069 (PMC13510525; doi:10.3390/biotech15030069)
Supplement: Supplementary file 1 [file biotech-15-00069-s001.zip › biotech-4400669-supplementary.pdf]

Supplementary Materials: Analysis of Quality Normative Requirements: Contributions to the Alignment and Standardization of Standards Applied in Health Laboratories

Saada L. C. Fernandez, Katharine V. S. Hodel, Renata Almeida de Souza, Hayna Malta-Santos, Camila Duarte Ferreira Ribeiro and Bruna A. S. Machado

Table S1. Definition of the terms used in comparative analyses of standards.

Term	Definition	Practical examples
**Equivalence**	Identical requirements in scope and intent	**Document control:** ISO 9001 (7.5), ISO 17025 (8.3), and ISO 15189 (4.3) require formal document control
**Alignment**	Compatible requirements, but with differences in depth or technical focus	**Risk management**: ISO 9001 calls for a risk-based approach (6.1), while ISO 17025 and 15189 require a more detailed assessment of operational and laboratory risks
**Same concept**	Same idea with a different approach	**Personnel competence:** ISO 9001 addresses competence in a general way (7.2), while ISO 17025/15189 detail technical requirements (qualification, revalidation, proficiency testing)
**Not evidenced**	Not found or not explicitly required in another standard	Validation of examination methods: strongly required in ISO 17025/15189, but not detailed in ISO 9001

Table S2. Key elements and description of principles of ISO 9001.

Key Elements	Principles
**Systemic Approach**	ISO 9001 emphasizes the importance of a systemic approach to quality management. When applying this standard, stakeholders are encouraged to consider all elements of their management system in an integrated manner, promoting a comprehensive understanding of the processes involved.
**Continuous Improvement**	The standard emphasizes the need for continuous improvement, encouraging the institution/area/department to regularly evaluate its processes and systems. This is essential to ensure that laboratory practices are always aligned with best practices and evolve according to changes in regulatory requirements in the health sector.
**Process Standardization**	ISO 9001 establishes specific requirements for documenting processes and procedures. This results in the standardization and monitoring of operational practices, which are particularly critical in laboratories, where consistency in methods and execution is essential for the validity and reliability of results.
**Customer Focus**	The standard emphasizes customer satisfaction. In the context of health laboratories, this means ensuring the accuracy and reliability of results, as well as effectively responding to the needs and expectations of customers, which may include researchers, health institutions, regulatory agencies, and funding bodies.
**Risk Management**	ISO 9001 introduces a risk-based approach. Applying this concept to laboratories means that laboratories must identify, assess, and address the risks associated with their activities. In the healthcare context, a risk-based mindset is vital to ensuring data integrity and the safety of all involved.
**Leadership Engagement**	The standard highlights the importance of leadership involvement in implementing and maintaining the QMS. This ensures a commitment to quality at all levels of the organization. The QMS comprises a set of organizational policies, processes, and procedures, and by leading "the effort" to implement it, leaders play a key role in creating an organizational culture that values quality, promotes excellence, and ensures compliance with applicable standards and regulations.
**Audits and Evaluations**	The standard requires regular audits and performance evaluations of the Quality Management System. These practices help identify opportunities for improvement and ensure compliance with the requirements of the standard. It also serves as an instrument for managing and monitoring the QMS in health laboratories.

Table S3. Key elements and description of principles of ISO/IEC 17025.

Key Elements	Principles
**Technical Competence**	The ISO/IEC 17025 standard requires laboratories to demonstrate technical competence in their areas of expertise. For laboratories in healthcare, this means ensuring that staff are properly qualified and trained, and that the methods and procedures used are aligned with best practices. Technical competence in laboratories refers to the ability of professionals and the laboratory as a whole to accurately and reliably perform the examinations, tests, and calibrations for which they are assigned. This competency covers several aspects, including technical knowledge, practical skills, experience, ability to interpret results, and adherence to quality procedures and standards.
**Impartiality**	The standard highlights the importance of impartiality in testing and calibration activities. In healthcare, impartiality ensures that results are not influenced by conflicting interests, guaranteeing the integrity and objectivity/clarity of the results.
**Consistency of Results**	ISO/IEC 17025 emphasizes the need for accurate and reliable results. When test results are consistent and reproducible over time, this increases confidence in the accuracy of the conclusions drawn from these data in healthcare laboratories.
**Metrological Traceability**	The standard requires laboratories to establish and maintain metrological traceability of measurements. In healthcare laboratories, this requires reference standards and accurately documenting the entire flow, ensuring data integrity.
**Control of Equipment and Environmental Conditions**	ISO/IEC 17025 requires the implementation of controls. In health-related activities, where small variations can have significant impacts, it is necessary to ensure the accuracy and reliability of laboratory results.
**Validation of Analytical Methods**	The standard addresses the validation of analytical methods, ensuring that the methods used in laboratories are fit for purpose and capable of producing reliable results. Validation is a dynamic process that must be conducted rigorously to ensure the quality of the results generated in laboratories.
**Documentation and Records**	The standard requires the maintenance of complete documentation and detailed records. In health-related laboratories, this documentation and record-keeping ensure the transparency, traceability, and reproducibility of the actions taken, data quality, and compliance.
**Measurement Uncertainty Assessment**	It is essential for laboratories that work in the health area to understand and report inconsistencies associated with results and the tolerable limits for each test; to this end, ISO/IEC 17025 guides laboratories in evaluating measurement uncertainty.

Table S4. Key elements and description of the principles of ISO 15189**.**

Key Elements	Principles
**Quality Management System**	The standard brings a structured approach to the process integration, management, and continuous improvement of the quality of laboratory products or services. It includes management and technical processes and reinforces the implementation of QMS in laboratories in the health service chain, where accurate results are the basis for decision-making and correct clinical conduct.
**Improvement management**	ISO 15189 highlights the need to implement a QMS that ensures compliance with technical standards, in addition to promoting a culture of continuous improvement. By emphasizing authority and accountability, the standard encourages the proactive identification of opportunities for improvement and the implementation of preventive and corrective measures.
**Standardized processes**	Harmonization and alignment in documenting activities in the standard contribute to comparing laboratory results, benefiting the medical community, patients, and stakeholders worldwide. This is critical in healthcare laboratories, where the accuracy and reliability of results are paramount. This highlights the importance of adapting requirements to meet the specific needs of laboratories in the health area while maintaining a focus on the quality and reliability of results.
**Quality assurance of results**	In ISO 15189, quality assurance for results and tests in clinical laboratories ensures the reliability and accuracy of the data generated, contributing to the integrity of the information. Participation in external proficiency assessment programs is a way to compare and evaluate laboratory activity. In this process, independent organizations send samples to different laboratories, which analyze and report the results. Comparison of results between laboratories provides insight into the accuracy and reliability of laboratory examinations.
**Critical analysis of results**	The standard involves a careful evaluation of the data generated by laboratory procedures, identifying references, controls, trends, discrepancies, and other relevant information.
**Pre-analytical, analytical, and post-analytical processes**	ISO 15189 establishes requirements for all stages of laboratory analysis, from patient identification at collection to communication of results. This interconnected, seamless operation strengthens compliance with regulatory requirements and the implementation of healthcare laboratory quality.

Table S5. Key elements and description of principles of NIT DICLA 035.

Key Elements	Principles
**Responsibilities**	The standard requires the definition of responsibilities and job descriptions for all hierarchies that work in the testing facility, which can also be set up in health laboratories. These responsibilities must be aligned with other activities developed, avoiding conflicts of interest. The definition of responsibilities promotes traceability and commitment to actions and functions in a health laboratory.
**Study Plan**	Before the start of each study, a documented plan should be made. According to NIT DICLA 035, this study plan must be checked for compliance with GLP principles and approved by the study director. The study plan in a laboratory is a document that details the approaches, methodologies, and procedures that are used when carrying out a project. This plan is formalized to ensure the efficiency, reliability, and validity of results in health laboratories.
**Testing methods**	All projects should refer to recognized methods, such as the OECD Test Guides, as well as the details of the experimental design, including a timeline of the study conducted in the laboratory, all methods, materials, and conditions; the type and frequency of laboratory activities, measurements, observations, and tests to be performed; and the statistical methods to be used. The health laboratory can act in the validation and understanding of the results of the project/investigation, especially considering the reproducibility, standardization, scientific transparency, and adequate interpretation of the results. The description of the methods also allows other interested parties to use the work as a starting point to search for other information or to develop new approaches.
**Study results**	According to the standard, each study must have a final report. When studies are short, a standardized final report can be made that also includes a specific extension of the study. In laboratories in the health area, a final report must also be prepared, as it organizes and summarizes all the stages of the study and documents and validates the efforts made to ensure the reliability of the data obtained.
**Storage and Retention of Records and Materials**	This item recommends the maintenance of documentation and materials that are part of the project, structured as files for a period usually determined by regulatory authorities. It includes the raw data, study plans, test and substance samples, references, specimens, and final reports of each trial. The proper storage and retention of records and materials in laboratories ensure integrity, traceability, and compliance with regulations, criteria, and standards. Effective record and material management meets regulatory requirements and contributes to operational efficiency and safety in the laboratory, with performance and health impacts.
**Storage and Retention of Records and Materials**	NIT DICLA 035 recommends keeping the documentation and materials of the project structured as files for a period usually determined by regulatory authorities. This includes the raw data, study plans, test and substance samples, references, specimens, and final

reports of each trial. The proper storage and retention of records and materials in laboratories ensure integrity, traceability, and compliance with regulations, criteria, and standards. Effective record and material management meets regulatory requirements and contributes to operational efficiency and safety in the laboratory, with performance and health impacts.
